# Induction of apoptosis by laminarin, regulating the insulin-like growth factor-IR signaling pathways in HT-29 human colon cells

**DOI:** 10.3892/ijmm.2012.1084

**Published:** 2012-08-02

**Authors:** HEE-KYOUNG PARK, IN-HYE KIM, JOONGKYUN KIM, TAEK-JEONG NAM

**Affiliations:** 1 Departments of Food Science and Nutrition; 2 Biotechnology, Pukyong National University, Nam-gu, Busan 608-737, Republic of Korea

**Keywords:** laminarin, caspase-3, insulin-like growth factor-IR signaling pathway

## Abstract

In recent years, algae have been highlighted as potential sources of anticancer agents. Laminarin is a molecule found in marine brown algae that has potentially beneficial biological activities. However, these activities have not been investigated. In the present study, we examined the effects of laminarin on HT-29 cells and analyzed its effect on the insulin-like growth factor (IGF-IR) signaling pathway. 3-(4,5-Dimethylthiazol-2-yl)-5-(3-carboxymethoxy-phenyl)-2-(4-sulfophenyl)-2H-tetrazolium (MTS) assays revealed that laminarin induced cell death in a dose-dependent manner. Western blotting showed that laminarin decreased mitogen-activated protein kinases (MAPK) and ERK phosphorylation. Decreased proliferation depended on IGF-IR, which was associated with the downregulation of MAPK/ERK. These results are important for understanding the roles of IGF-IR in colon cancer cell tumorigenesis, and suggest that laminarin shows activity against human colon cancer.

## Introduction

Asian countries consume a traditional diet high in seaweed (1). Brown seaweeds are a potential source of bioactive ingredients. They also contain large amounts (∼40% of dry matter) of polysaccharides, which are considered dietary fibers (2,3). These polysaccharides include laminarin, fucoidan, and alginates. Of these, laminarin is a storage glucan found in brown algae (4), and is composed of β-glucan (β1–3, β1–6-glucan) (5). Due to these characteristics, laminarin is assumed to have biological activities similar to those of other glucans. Glucans are highly functional materials that are FDA-approved for lowering cholesterol. They have been shown to stimulate immunity, and to have antitumor effects and antibacterial activity (6–8). Moreover, they have been studied extensively for their immunological and pharmacological effects. However, the biological activities of laminarin have yet to be investigated. To evaluate its potential inhibition of colon cancer, we evaluated the effects of laminarin *in vitro*.

Apoptosis is important in the normal development and differentiation of a wide variety of tissues. Apoptosis is characterized by several unique features, including cell shrinkage, chromatin condensation, DNA fragmentation, the cell surface expression of phosphatidylserine, and membrane blebbing (9,10). Predominantly, apoptosis may be initiated in two ways: by an intrinsic (mitochondrial-mediated) or by an extrinsic (death receptor-mediated) pathway (11–13). Each pathway results from the activation of caspases and ultimately leads to apoptosis. In the latter case, transmembrane death receptors are involved and the apoptotic signal occurs by the interaction between the ligands and the death receptor. A wide range of physical and chemical changes of mitochondrial integrity may be triggered by stimulating the intrinsic pathway of apoptosis (11–13). However, most cancer cells block apoptosis, allowing the survival of malignant cells, despite genetical and morphological changes. Fas and FasL are apoptosis-inducing members of the TNF-cytokine family. Fas activation by FasL and its receptor FADD activate caspases-3, -8 and -9, leading to apoptosis (14–16). Thus, we aimed to determine whether laminarin inhibits cell growth and induces apoptosis in colon cancer cells.

Insulin-like growth factor-I receptor (IGF-IR) is significant in cell growth, differentiation, and survival (17). Overexpression of IGF-IR and related proteins results in cancer cell proliferation and survival. Thus, IGF-IR is involved in malignant transformation (18–20). Therefore, IGF-IR and related proteins are attractive anticancer targets.

In the present study, we aimed to determine whether laminarin induced apoptosis by molecular mechanisms involving IGF-IR and cell death pathways. We examined the manner in which laminarin regulates HT-29 cells, and assessed its effect on the Fas and IGF-IR signaling pathways. The results showed that activation of Fas-induced apoptosis blocks the IGF-IR pathway.

## Materials and methods

### Cell culture

Human colon adenocarcinoma cells (ATCC HTB-38) and rat small intestine epithelial cells (IEC-6, ATCC CRL-1592) were obtained from the American Type Culture Collection (Rockville, MD, USA). Cells were maintained in a humidified 5% CO_2_, 95% air, 37°C environment in RPMI-1640. DMEM was supplemented with penicillin/streptomycin (P/S), and HT-29 and IEC-6 cell cultures were supplemented with 10% fetal bovine serum (HyClone, Inc., South Logan, UT, USA). Cells in the exponential phase were used.

### Cell viability

Laminarin (L-9634) was purchased from Sigma-Aldrich (St. Louis, MO, USA). The effects of various laminarin concentrations on the cell proliferation of HT-29 and IEC-6 cells were determined colorimetrically after 24 h using the 3-(4,5-dimethylthiazol-2-yl)-5-(3-carboxymethoxyphenyl)-2-(4-sulfophenyl)-2H-tetrazolium (MTS) assay with Cell Titer 96^®^ AQueous One Solution Reagent (Promega, Madison, WI, USA). Cells were seeded onto 96-well plates at 2×10^4^ cells/well in 100 *μ*l medium and incubated for 24 h. Attached cells were maintained in serum-free medium (SFM) for 12 h, followed by laminarin treatment (0-5 mg/ml) for 24 h. Subsequently, cells were incubated with MTS solution at 37°C for 30–60 min and the absorbance of each well was measured at 490 nm using a microplate reader. The OD490 values of the control cells were designated as 100%.

### Caspase activity

Caspase activities were measured using caspase-3 substrate I (Ac-DEVD-pNA; 235400), caspase-8 substrate I (Ac-IETD-pNA) and caspase-3 inhibitor [Z-D(Ome)-E-(Ome)-V-D(OMe)-FMK; 368057; Calbiochem, San Diego, CA, USA]. Cells were seeded in culture dishes and grown to 60% confluence. These cells were treated with 50 *μ*M caspase inhibitor for 1 h and laminarin for 24 h, after which caspase lysis buffer (2.5 mM HEPES, pH 7.5, 5 mM EDTA, 2 mM DTT, 0.1% CHAPS) was added. A total of 100 *μ*g protein/100 *μ*l was collected, and 2 *μ*l of the substrate was added to the wells. Cells were incubated with a caspase substrate in a shaking incubator at 37°C for 4 h. The absorbance at 405 nm was then determined using an ELISA plate reader.

### Western blotting

To prepare whole-cell extracts, cells were washed with PBS and suspended in extraction buffer (50 mM Tris-HCl, pH 7.4, 150 mM NaCl, 0.25% Na-deoxycholate, 1% NP-40, and 1 mM EGTA) containing protease inhibitors (1 mM Na_3_VO_4_, 1 *μ*g/ml aprotinin, 1 *μ*g/ml pepstatin, 1 *μ*g/ml leupeptin, 1 mM NaF, and 1 mM PMSF) on ice. The extracts were centrifuged at 12,000 rpm for 10 min and the supernatant was used in western blotting. Boiling sample buffer (50 *μ*g/ml) was added to the total cell lysate and the samples were boiled for 10 min at 100°C. Proteins were separated in 7.5–15% SDS-PAGE gels and transferred to PVDF membranes (Millipore, Billerica, MA, USA). Membranes were blocked for 1 h at room temperature in blocking buffer [1% bovine serum albumin (BSA) in TBS-T]. Blots were probed with primary antibodies (1:1,000 in 1% BSA/TBS-T) for 18 h at 4°C. The membranes were then washed twice for 15 min in TBS-T. The secondary antibody was a horseradish peroxidase (HRP)-conjugated goat anti-mouse or rabbit antibody (1:10,000 in 1% BSA/TBS-T). Signal bands were detected using an enhanced chemiluminescence western blotting kit (Amersham Biosciences, Piscataway, NJ, USA).

### Statistical analysis

Multiple mean values were compared by analysis of variance using the SPSS software (SPSS, Inc., Chicago, IL, USA). Values were presented as the means ± standard deviation. P<0.05 was considered statistically significant. Values in Fig. 3, indicated with the letters a-d were significantly different according to the Duncan’s multiple range test.

## Results

### Laminarin reduces the proliferation of HT-29 cells

**Figure 1:**
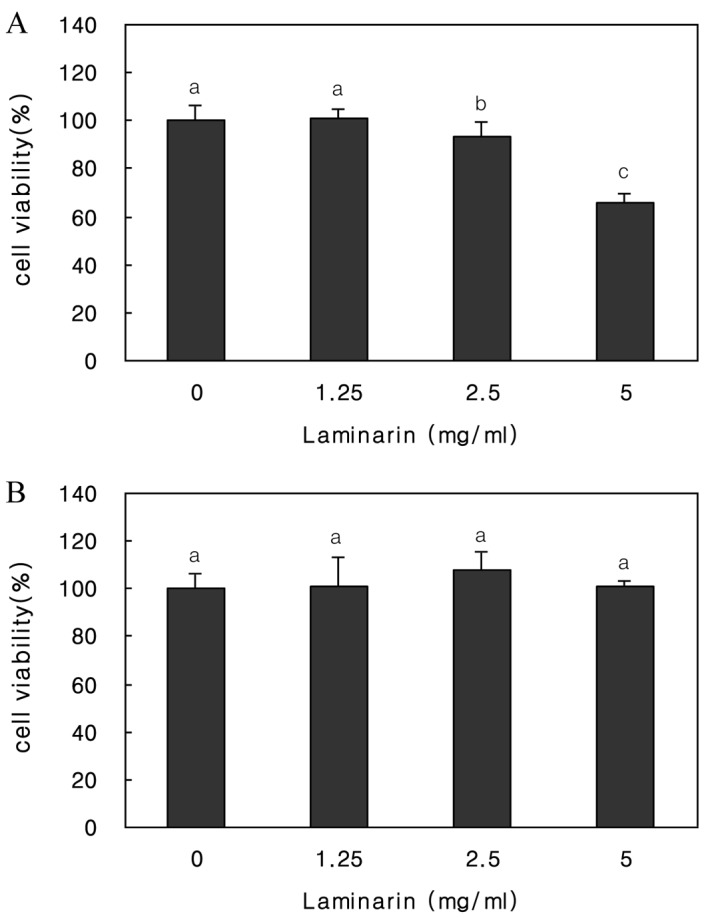
Effect of laminarin on HT-29 cell proliferation. (A) Effect of laminarin treatment on the growth of HT-29 colon cancer cells. Cells were treated with varying concentrations of laminarin (0, 1.25, 2.5, 5 mg/ml) for 24 h. (B) Laminarin toxicity to IEC-6 intestinal epithelial cells.

We determined the effect of 24-h laminarin treatment (0, 1.25, 2.5 and 5 mg/ml) on the viability of HT-29 and IEC-6 cells by MTS assay (Fig. 1). Laminarin treatment decreased the proliferation of HT-29 cells in a dose-dependent manner. Exposure to 5 mg/ml laminarin inhibited cell growth by 60%. By contrast, IEC-6 cells were unaffected. Moreover, no toxicity to either cell type was detected.

### Laminarin induces morphological changes of cells

**Figure 2:**
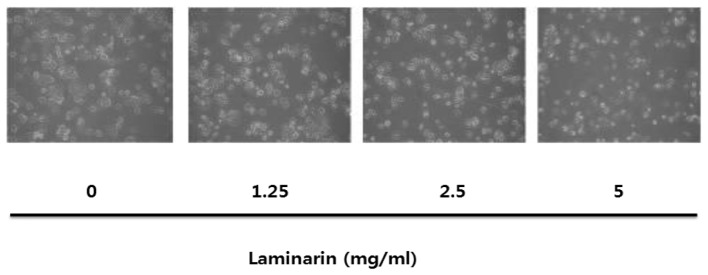
Morphological changes in HT-29 cells after laminarin treatment. After 24 h of laminarin treatment (0–5 mg/ml), cells were observed under an optical microscope. Magnification, x200.

The effect of laminarin on cell and nuclear morphology was determined using an MTS assay and light microscopy (Fig. 2). The survival of HT-29 cells was reduced in a laminarin concentration-dependent manner. Cells also decreased in size in a laminarin concentration-dependent manner.

### Laminarin-induced apoptosis is mediated by caspase-3

**Figure 3:**
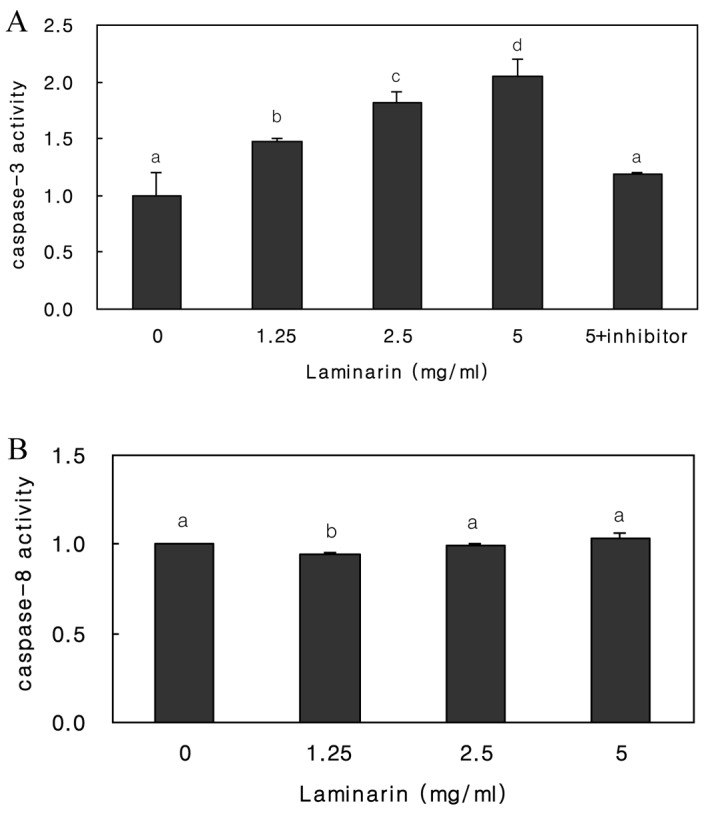
(A) Dose-dependent activation of caspase-3 by laminarin treatment in HT-29 cells. Laminarin was added at concentrations of 0, 1.25, 2.5 and 5 mg/ml. Caspase-3 inhibitor was added at a concentration of 5 mg/ml. (B) Laminarin did not affect caspase-8 expression in HT-29 cells. Values represent means ± SD; P<0.05 was determined by ANOVA. Values with different letters are significantly different according to the Duncan’s multiple range test.

To determine which caspases are activated by laminarin, we identified laminarin-induced enzyme activities (Fig. 3). A significant increase was found in the level of caspase-3, but not caspase-8. We examined caspase-3 activation after laminarin treatment in the presence of a caspase-3 inhibitor. The caspase-3 inhibitor completely blocked caspase-3 activity, suggesting that laminarin activates caspase-3, but not caspase-8.

### Laminarin induces the expression of apoptosis-related proteins

A wide variety of signaling molecules are combined with cell-surface receptors. Fas (CD95, APO-1), a member of the tumor necrosis factor family, is a cell death receptor that plays a key role in the regulation of homeostasis (21).

**Figure 4:**
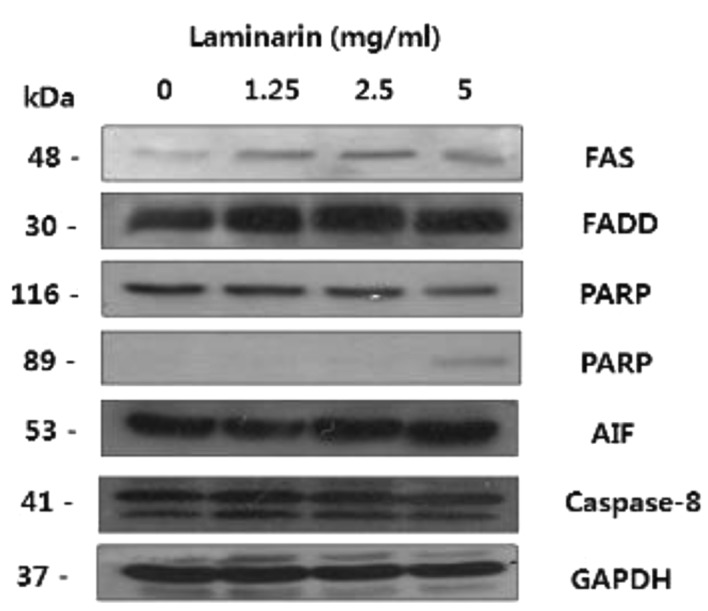
Laminarin affects the expression of the apoptosis-related proteins FAS, FADD, PARP, AIF, and caspase in HT-29 cells. Laminarin induced DISC formation in HT-29 cells. The cells were treated with laminarin (0–5 mg/ml) for 24 h.

Fas and the Fas receptor induce the activation of members of the caspase family, and subsequently the cleavage of markers of apoptosis such as poly (ADP-ribose) polymerase (PARP) (22). This signaling cascade is known as the Fas signaling pathway. Following laminarin treatment, an increase was observed in the expression of FAS and FADD (Fig. 4). We previously reported that laminarin treatment caused caspase-3 activation and PARP cleavage. These results suggest that laminarin induced apoptosis via the Fas signaling pathway.

### Laminarin induces the expression of IGF-IR signaling pathway-related proteins

**Figure 5:**
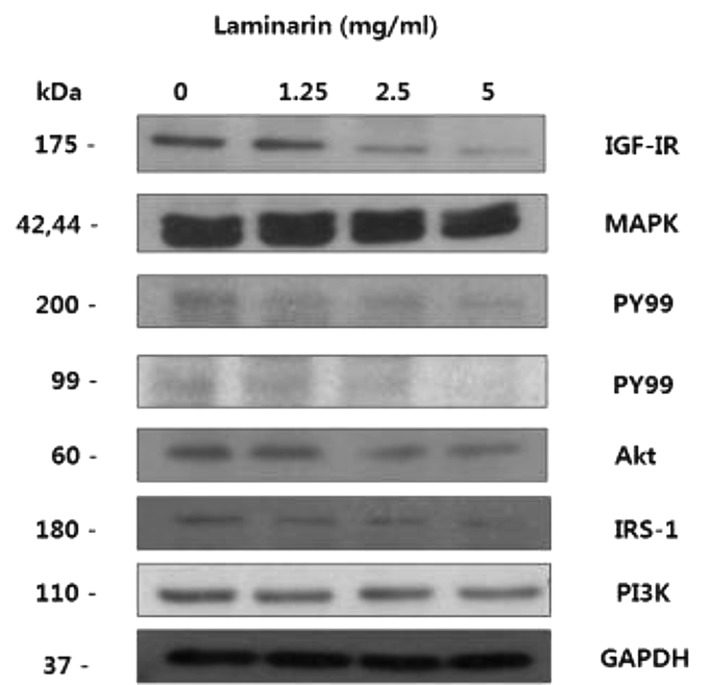
Laminarin affects the protein expression of the IGF-IR signaling pathway in HT-29 cells. Cells were treated with laminarin (0–5 mg/ml) for 24 h. Proteins were visualized by western blotting.

Laminarin induced apoptosis via the Fas signaling pathway. Cell death signaling mechanisms and cell growth were also affected by laminarin. The growth-inhibitory effect of laminarin was associated with changes in the expression of proteins involved in the IGF-IR pathway in HT-29 cells (Fig. 5). Signaling pathways activated by IGF-IR include the mitogen-activated protein kinases (MAPK) and phosphatidylinositol 3-kinase (PI3K) pathways (23). This signaling is controlled by the IGF-binding protein (IGFBP). A decreased expression of the IGF-IR and downstream signaling proteins such as PI3K, PY99, Akt and MAPK IRS-1 inhibits events in cancer. These results suggest that laminarin may inhibit cancer development by regulating the IGF-IR pathway.

### IGF-IR proteins are inhibited by Fas-mediated caspase activation

We demonstrated that laminarin induces apoptosis in a Fas-mediated manner. In addition, we found that laminarin downregulates IGF-IR-related proteins.

**Figure 6:**
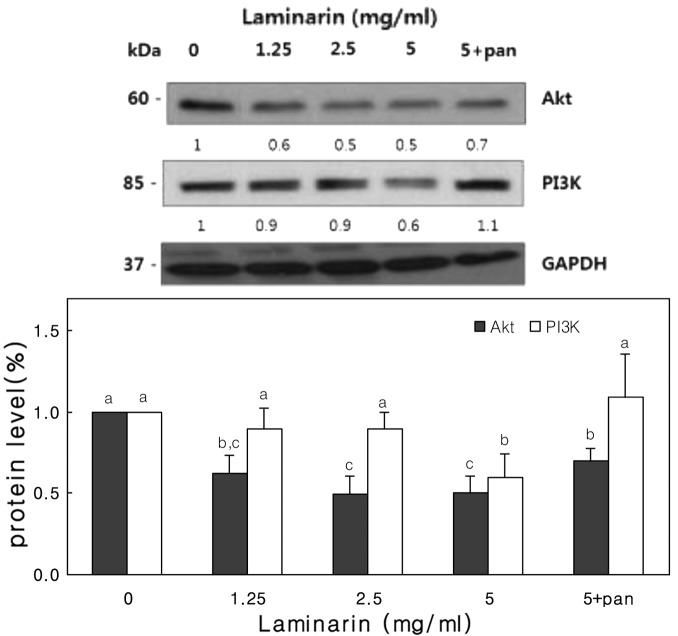
Effects of laminarin on the pancaspase inhibitor-induced recruitment of PI3K and Akt in HT-29 cells. Laminarin was added at concentrations of 0, 1.25, 2.5 and 5 mg/ml. HT-29 cells were treated for 1 h with or without the pancaspase inhibitor [Z-VAD(Ome)-FMK; 5 mg/ml]. Each bar shows the quantitative analysis of immunoblots.

Therefore, we determined whether Fas-induced apoptosis blocks the IGF-IR pathway. In HT-29 cells, pancaspase inhibitor treatment suppressed caspase activation. Results of the western blot analysis showed that inhibitor treatment resulted in decreased caspase-3 levels (data not shown) and the recruitment of PI3K and Akt (Fig. 6). Thus, the IGF-I pathway is involved in laminarin-induced apoptosis.

## Discussion

The anticancer effect of seaweed has been the focus of many recent studies. Seaweed contains large amounts (∼40% of the dry matter) of polysaccharides, primarily laminarin, fucoidan, and alginates. In the present study, we found that laminarin inhibits HT-29 cell growth by decreasing cell proliferation and inducing apoptosis.

To the best of our knowledge, we have provided the first evidence that laminarin regulates apoptosis and the IGF-IR-related protein expression. When HT-29 cells were incubated with laminarin, cell viability was decreased. HT-29 cells treated with laminarin exhibited morphological changes; cells decreased in size in a laminarin concentration-dependent manner.

FasL and its receptor FADD are adapter molecules required for Fas-mediated apoptosis (24,25). Laminarin regulated Fas and FADD protein levels, suggesting that it induces Fas-mediated apoptosis. It also increased the expression of Fas and FADD, which in turn induced the activation of members of the caspase family (26,27). Caspases play a key role in cell death-related apoptosis. We analyzed caspase activation during laminarin-induced apoptosis using caspase substrates. In HT-29 cells, we detected a significant increase in the level of caspase-3, but not caspase-8. Caspase-8 is an initial caspase in apoptosis and is essential to the Fas-mediated apoptosis pathway (28,29). Previous reports have shown that caspase-8 may induce apoptosis independent of Fas. In their study, Feng *et al* (30) reported that Fas-FADD oligomerization is able to trigger a novel caspase-8-independent pathway.

IGF-I signaling plays a role in cancer development and progression (31,32). Remacle-Bonnet *et al* (33) showed that IGF-I protected cancer cells against apoptosis. The mechanisms by which IGF-I and IGF-I receptors interact with cell death pathways remain unclear. Therefore, it is important to elucidate the relationships between IGF-I and IGF-I receptors and apoptotic pathways.

**Figure 7:**
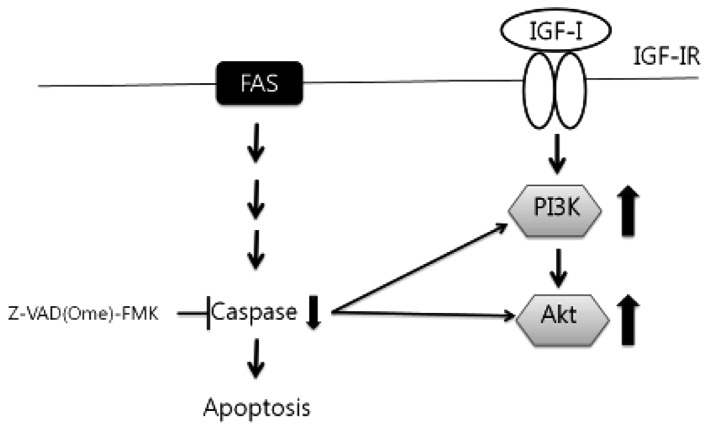
Model of Fas-induced apoptosis of inhibition of the IGF-IR-associated proteins Akt and PI3K. Z-VAD(Ome)-FMK inhibits caspase activity, which is activated through Fas, and increases Akt and PI3K expression.

We examined the effect of laminarin on the IGF-IR pathway. A decreased expression of IGF-IR and downstream signaling proteins inhibits events in cancer. These results suggest that laminarin inhibits cancer by regulating the IGF-IR pathway. As shown in Figs. 6 and 7, a pancaspase inhibitor suppressed caspase activation in HT-29 cells. This inhibition affected the expression of IGF-I receptor pathway-related proteins. Therefore, we demonstrated that the activation of Fas-induced apoptosis blocks the IGF-IR pathway.

These data suggest that laminarin has the potential to be used as an anticancer agent. Recently, studies have reported anticancer effects of seaweeds (34,35). However, to the best of our knowledge, this is the first report of laminarin activity against human colon cancer cells. Therefore, the regulation of these two pathways may be important for the treatment of human colon cancer and serve as a novel target of anticancer supplements and drugs.
